# Evaluation of ^18^F-FDG PET/CT images acquired with a reduced scan time duration in lymphoma patients using the digital biograph vision

**DOI:** 10.1186/s12885-020-07723-2

**Published:** 2021-01-14

**Authors:** Manuel Weber, Walter Jentzen, Regina Hofferber, Ken Herrmann, Wolfgang Peter Fendler, Christoph Rischpler, Lale Umutlu, Maurizio Conti, Pedro Fragoso Costa, Miriam Sraieb, David Kersting

**Affiliations:** 1 Department of Nuclear Medicine, University of Duisburg-Essen, University Hospital Essen, Hufelandstrasse 55, 45122 Essen, Germany; 2 University of Duisburg-Essen and German Cancer Consortium (DKTK)-University Hospital, Essen, Germany; 3 Department of Department of Diagnostic and Interventional Radiology and Neuroradiology, University Hospital Essen, Essen, Germany; 4 Siemens Medical Solutions USA, INC, Erlangen, Germany

**Keywords:** PET/CT, FDG, Image quality, Silicon photomultiplier, Lymphoma

## Abstract

**Background:**

The superior accuracy and sensitivity of ^18^F-FDG-PET/CT in comparison to morphological imaging alone leads to an upstaging in up to 30% of lymphoma patients. Novel digital PET/CT scanners might enable to reduce administered tracer activity or scan time duration while maintaining diagnostic performance; this might allow for a higher patient throughput or a reduced radiation exposure, respectively. In particular, the radiation exposure reduction is of interest due to the often young age and high remission rate of lymphoma patients.

**Methods:**

Twenty patients with (suspected) lymphoma (6 for initial staging, 12 after systemic treatment, 2 in suspicion of recurrence) sequentially underwent ^18^F-FDG-PET/CT examinations on a digital PET/CT (Siemens Biograph Vision) with a total scan time duration of 15 min (reference acquisition protocol) and 5 min (reduced acquisition protocol) using continuous-bed-motion. Both data sets were reconstructed using either standalone time of flight (TOF) or in combination with point spread function (PSF), each with 2 and 4 iterations. Lesion detectability by blinded assessment (separately for supra- and infradiaphragmal nodal lesions and for extranodal lesions), lesion image quantification, and image noise were used as metrics to assess diagnostic performance. Additionally, Deauville Score was compared for all patients after systemic treatment.

**Results:**

All defined regions were correctly classified in the images acquired with reduced emission time, and therefore, no changes in staging were observed. Lesion quantification was acceptable, that is, mean absolute percentage deviation of maximum and peak standardized uptake values were 6.8 and 6.4% (derived from 30 lesions). A threefold reduction of scan time duration led to an increase in image noise from 7.1 to 11.0% (images reconstructed with 4 iterations) and from 4.7 to 7.2% (images reconstructed with 2 iterations). No deviations in Deauville Score were observed.

**Conclusion:**

These results suggest that scan time duration or administered tracer activity can be reduced threefold without compromising diagnostic performance. Especially a reduction of administered activity might allow for a lower radiation exposure and better health economics. Larger trials are warranted to confirm our results.

**Supplementary Information:**

The online version contains supplementary material available at 10.1186/s12885-020-07723-2.

## Background

The 2014 Lugano Classification recommends performing ^18^F-FDG-PET/CT in lymphoma patients for interim staging and after the completion of chemotherapy for the evaluation of treatment response. Accuracy in terms of sensitivity has been shown to be higher than in standalone morphological imaging, leading to an upstaging in up to 30% of patients, especially in a subcohort of frequently FDG-avid lymphoma subtypes [1]. Additionally, interim staging ^18^F-FDG-PET/CT can predict survival in lymphoma patients after systemic treatment with combined chemo- and immunotherapy [2].

The two most relevant caveats in the imaging of lymphoma patients are (a) the high prevalence of brown adipose tissue bearing the risk of false-positive results [3, 4] and (b) the often small size of nodal lesions potentially leading to false-negative results [5]. These factors do not only place stringent requirements on the medical imaging specialists, but also on the imaging devices, image acquisition protocols, and image reconstruction algorithms. To ensure optimal image quality for tumor imaging, the EANM procedure guidelines on ^18^F-FDG-PET/CT recommend starting the scan acquisition 60 min after the intravenous administration of ^18^F-FDG and a patient-specific optimization of administered tracer activity. The necessary ^18^F-FDG activity is calculated based on patient weight, scanning device, and emission time [6]. Typically, about 3 MBq/kg bodyweight of ^18^F-FDG are administered when using an emission time of about 3 min per bed position [7].

With the advent of a new generation of silicon photomultiplier-based, so called digital PET/CT devices a reduction of the injected amount of ^18^F-FDG appears feasible due to the higher detector sensitivity and improved coincidence timing resolution [7]: For example, phantom studies have shown that a reduction of acquisition time up to a factor of six is possible while maintaining a high diagnostic performance [8, 9]. Additionally, a threefold reduction in acquisition time duration (which is approximate to a reduction in administered activity by the same factor) only led to changes in tumor stage in a small fraction of oncological patients [10]. This finding has considerable implications:

The implementation of a low-activity acquisition protocol would lead to a reduction in radiation exposure for patients and medical staff. This low-activity regimen would be particularly beneficial for lymphoma patients, who are often young and have a high rate of long-term remission [11]. Additionally, lower activity requirements/scanning times would enable PET centers to perform more exams per day and optimize their cost efficiency.

We therefore aimed to evaluate the feasibility of a threefold reduction in scan time duration in lymphoma patients undergoing ^18^F-FDG-PET/CT without compromising diagnostic performance. As a reduction of emission time correlates to a reduction in administered activity by the approximately the same factor [10, 12], our results would advocate for the use of a low-activity protocol.

## Methods

### Patient population and preparation

Twenty consecutively enrolled lymphoma patients (5 with Hodgkin lymphoma, 14 with Non-Hodgkin lymphoma, 1 with high clinical suspicion of lymphoma) undergoing ^18^F-FDG PET examination (on clinical indication) were included. In 6 of these the examination was performed for initial staging, in 12 after systemic treatment and in the 2 remaining patients for suspicion of recurrence.

Detailed patient and imaging characteristics are provided in Supplemental Table S1. Mean patient age (range) was 50 (23–84) years and mean patient weight (range) was 81 (47–130) kg. Following joint EANM procedure guidelines for ^18^F-FDG PET/CT in tumor imaging, a mean±standard deviation (SD) activity of 340±72 MBq (corresponding to 4.2±0.4 MBq/kg bodyweight) ^18^F-FDG was injected intravenously. PET/CT data were acquired after a mean±SD time interval of 73±11 min.

### Image acquisition

All examinations were performed on a digital Biograph Vision PET/CT system (Siemens Healthcare; Erlangen, Germany), whose imaging properties have recently been assessed using ^18^F [7]. The scan area comprised whole-body PET/CT from mid-thigh to skull base. Image acquisition started with a whole-body spiral CT in full-dose technique using automatic tube current and tube voltage adjustments (Care Dose 4D, quality reference 160 mAs, CARE kV, quality reference 120 kV). These data were used for attenuation correction and anatomical correlation. Subsequently, two PET scans were applied in continuous-bed-motion mode.

The reference (or clinical standard) scan was acquired first and lasted approximately 15 min, the reduced scan was acquired subsequently with an emission time of about 5 min. We chose an approximate threefold reduction of emission time based on previously published in-vivo and phantom studies by other groups and an optimization study by our group performed on the same PET/CT system using an abdominal phantom under conditions observed in lymphoma imaging [10, 13, 14]. More precisely, in the phantom study our group has demonstrated that the optimized step-and-shoot emission time was approximately 60 s/bed (or 2.19 mm/s in continuous-bed-motion table speed) in association with appropriate image reconstruction algorithms (see below). Of note, the conversion from step-and-shoot emission time per bed (*t*_bed_) to continuous-bed-motion table speed (*v*_table_) was based on manufacturer recommended equivalence settings using an axial field of view (FOV) of 263 mm (or *v*_table_ = 0.5 FOV / *t*_bed_).

More specifically, our clinical standard protocol comprised three regions: two non-abdomen regions (ranging from the skull-base to the upper abdomen and from the lower abdomen to mid-thigh) and an abdomen region. For the reference acquisition protocol, the continuous-bed-motion table speed (equivalent step-and-shoot emission time per bed position, approximate scan length of about 30 cm) for the non-abdomen regions and within abdomen region was 1.5 mm/s (88 s/bed) and 0.8 mm/s (164 s/bed), respectively. For the reduced acquisition protocol, the continuous-bed-motion table speeds were 2.2 mm/s (60 s/bed) and 4.1 mm/s (32 s/bed) within the abdomen and non-abdomen region, respectively. This translates to a reduction of the scan time duration exactly by a factor of 2.75 or approximately a threefold reduction in scan time duration.

### Image reconstruction

The diagnostic CT images were reconstructed iteratively with a convolution kernel I30f (SAFIRE level of 3). The reconstructed CT slice thickness and the transversal voxel size was 3.0 mm and 1.5× 1.5 mm^2^, respectively. Based upon our previously conducted phantom-based optimization study images [13, 14] were reconstructed using the three-dimensional ordinary Poisson ordered-subset expectation maximization (OSEM) algorithm, either with standalone time of flight (TOF) approach or with combined TOF and point spread function (PSF). For both acquisition protocols, 4 image sets were reconstructed: TOF and TOF+PSF, each with 2 iterations (5 subsets) or 4 iterations (5 subsets). The reconstructed images had a voxel size of 3.3× 3.3× 3.0 mm^3^ and were smoothed with an isotropic Gaussian post-reconstruction filter of 4 mm. The estimated reconstructed PET spatial resolution (expressed as the full-width-at-half maximum) was 5.4 mm and 4.9 mm for TOF- and TOF+PSF-reconstructed images, respectively [13]. The resulting 4 images (reconstructed for each patient and each acquisition protocol) are referred to OSEM-TOF (2i), OSEM-TOF (4i), OSEM-TOF+PSF (2i), and OSEM-TOF+PSF (4i).

### Image analyses

Pseudonymized PET/CT data were analyzed by a central reader blinded to any clinical information in random order on a per-region basis. Based on the Ann-Arbor Classification, three types of regions based on lesion location were defined [1], which are: supradiaphragmal nodal lesions, infradiaphragmal nodal lesions, extranodal lesions. Subsequently, for each region, maximum and peak standardized uptake values (SUVmax and SUVpeak) were measured and its lesion size (short diameter for nodal lesions, long diameter for non-nodal lesion) were determined for the lesion with the highest tracer uptake. The resulting SUV ratios were further categorized in terms of SUVmax showing tumors with faint (SUVmax≤5), moderate (5<SUVmax< 10), and high uptake (SUVmax≥10). In addition, tumor stage according to the Ann-Arbor Classification and Deauville Score for patients after systemic treatment were assessed.

### Metrics for diagnostic performance

Three metrics were used to evaluate the diagnostic performance. Primary endpoint was the accuracy of the per-region detectability in the images acquired with the reduced protocol. To this end, images reconstructed with the same image reconstruction algorithm, but acquired with standard emission time duration, were set as reference image. Subsequently, the fraction of correctly classified tumor regions and subsequent changes in Ann-Arbor stage were assessed.

Secondary endpoints were the precision in image quantification and image noise. The former was obtained by calculating the ratio between SUVmax (SUVpeak) of FDG-avid tumors in the reduced and reference acquisition protocol series for each of the respective image reconstruction algorithms. Image noise was assessed using the liver’s activity distribution and is defined as the ratio of the standard deviation of SUV to the mean SUV in healthy liver tissue that were obtained by placing a spherical volume of interest with 3 cm in diameter in the lower right liver lobe [15, 16].

## Results

### Detectability

**Fig. 1:**
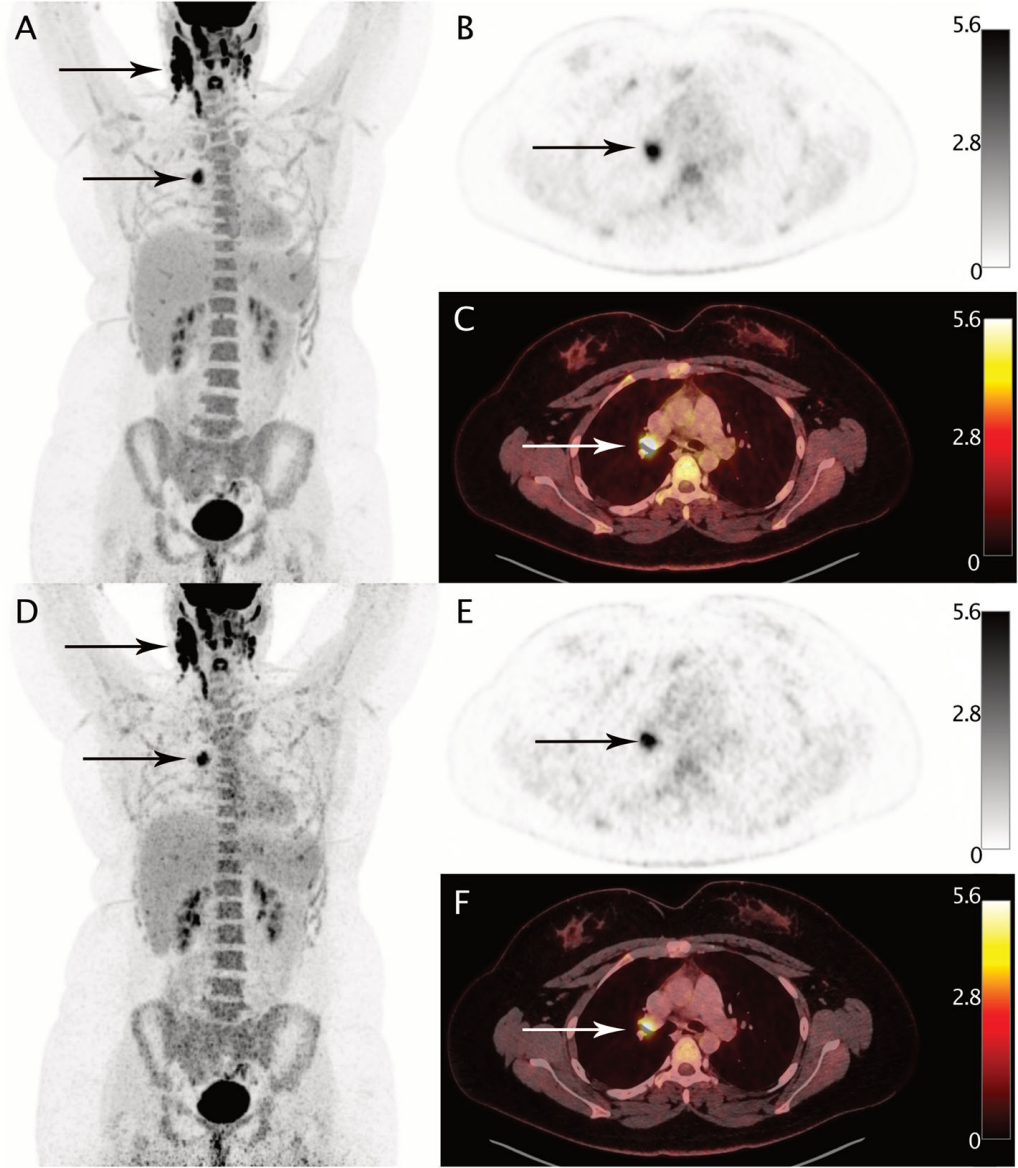
A 25-year-old patient with biopsy-proven lymphoma undergoing ^18^F-FDG-PET/CT before treatment. Panels **a**, **b**, and **c** show images acquired with the reference acquisition protocol, panels **c**, **d**, and **e** show image acquired with reduced emission time, all reconstructed with OSEM-PSF+TOF (4i). Axial slices (**b**, **c**, **c**, and **f**) and maximum intensity projections (**a** and **d**) reveal vital lymphoma manifestations in cervical and mediastinal lymph nodes. All lesions visible with standard acquisition protocol were also detectable after a threefold reduction of scan time duration. Values left to the color bares are in SUV units

As assessed by the reference protocol, 9/20 patients (45%) were PET-positive. Of these, 1/20 patients was staged as Ann-Arbor I, 4/20 were staged as Ann-Arbor II, and 2/20 each as Ann-Arbor III and IV (the latter with adrenal and bone involvement). Using images acquired with the standard protocol as reference, 60/60 regions (100%) and 12/12 (100%) regions with at least one tumor lesion were correctly classified in the reduced protocol (Supplemental Tables S2 and S3). All defined regions were correctly classified in the images acquired with reduced scan time duration, and therefore, no changes in staging were observed. In addition, no differences regarding the lesion detectability were observed between the different reconstruction algorithms. Figure 1 shows images acquired with standard vs. reduced acquisition time of patients, using OSEM-TOF+PSF (4i) exemplary.

### Image quantification

**Fig. 2:**
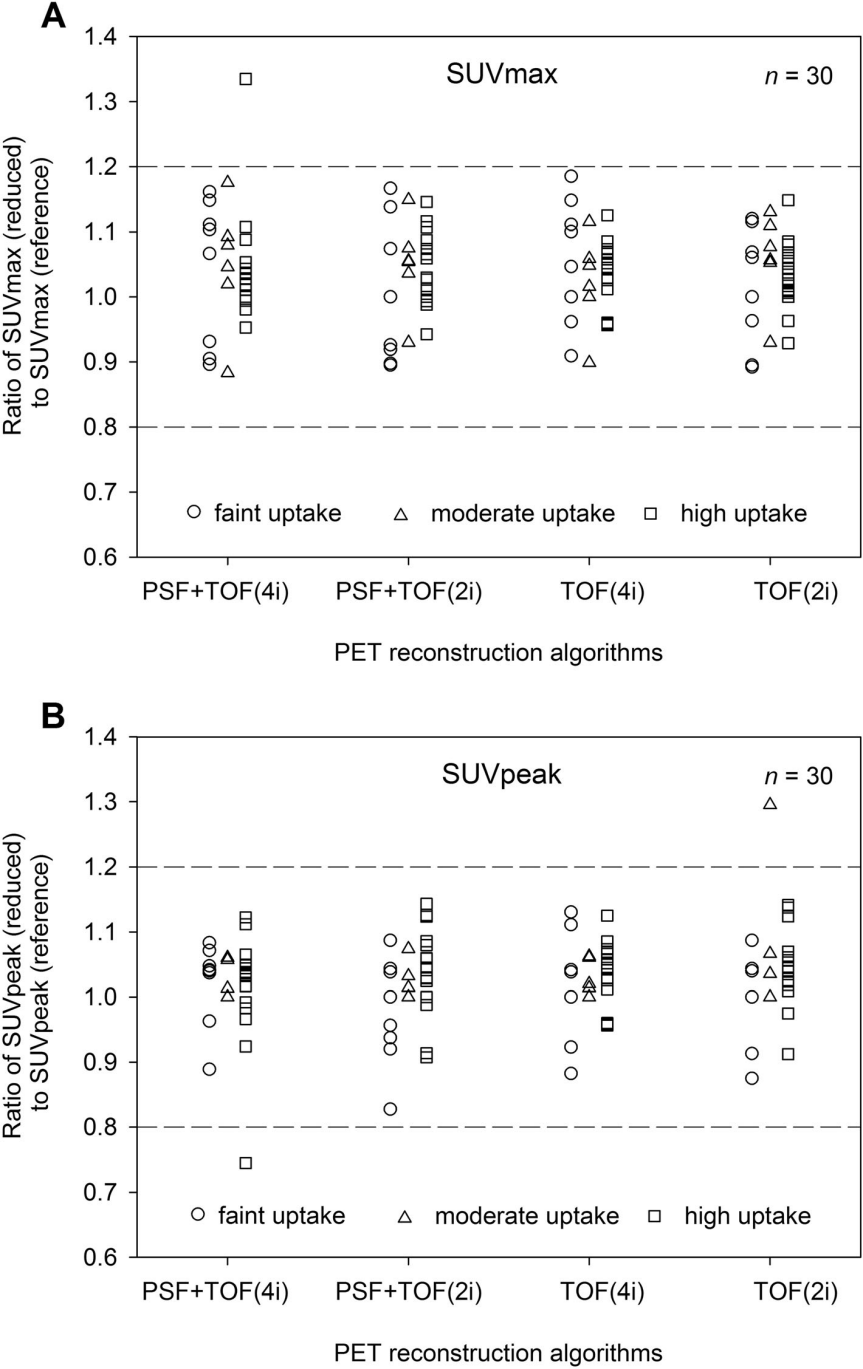
Dot plots showing the ratio between lesion SUVmax (panel **a**) and SUVpeak (panel **b**) between images acquired with reference vs. reduced acquisition protocol. Quantitative assessment was performed separately for lesions with faint (circles, SUVmax≤5), moderate (triangles, 5<SUVmax< 10)), and high uptake (squares, SUVmax ≥ 10). Dashed lines indicate the maximum tolerated deviation of ±20%

Figure 2 illustrates the ratio of SUVmax and SUVpeak, separately for lesions with faint, moderate, and high uptake. An absolute difference in SUVs of less than 20% was observed between images acquired with reduced vs. standard protocol. The error margin of ±20% was considered acceptable in this study, which is similar to the mean percentage difference in lesion SUVmax for different scanners at different locations [17]. Across all measured lesions, the mean absolute percentage deviation for SUVmax (SUVpeak) was 7.5% (8.4%), 6.8% (5.7%), 6.5% (5.3%), and 6.2% (6.2%) for OSEM-TOF+PSF (4i), OSEM-TOF+PSF (2i), OSEM-TOF (4i), OSEM-TOF (2i), respectively. In the population after systemic treatment (*n*=12), Deauville Score was 1 in four patients, 2 in three patients, 4 in three patients and 5 in two patients. No deviations in Deauville Score were observed.

### Image noise

**Fig. 3:**
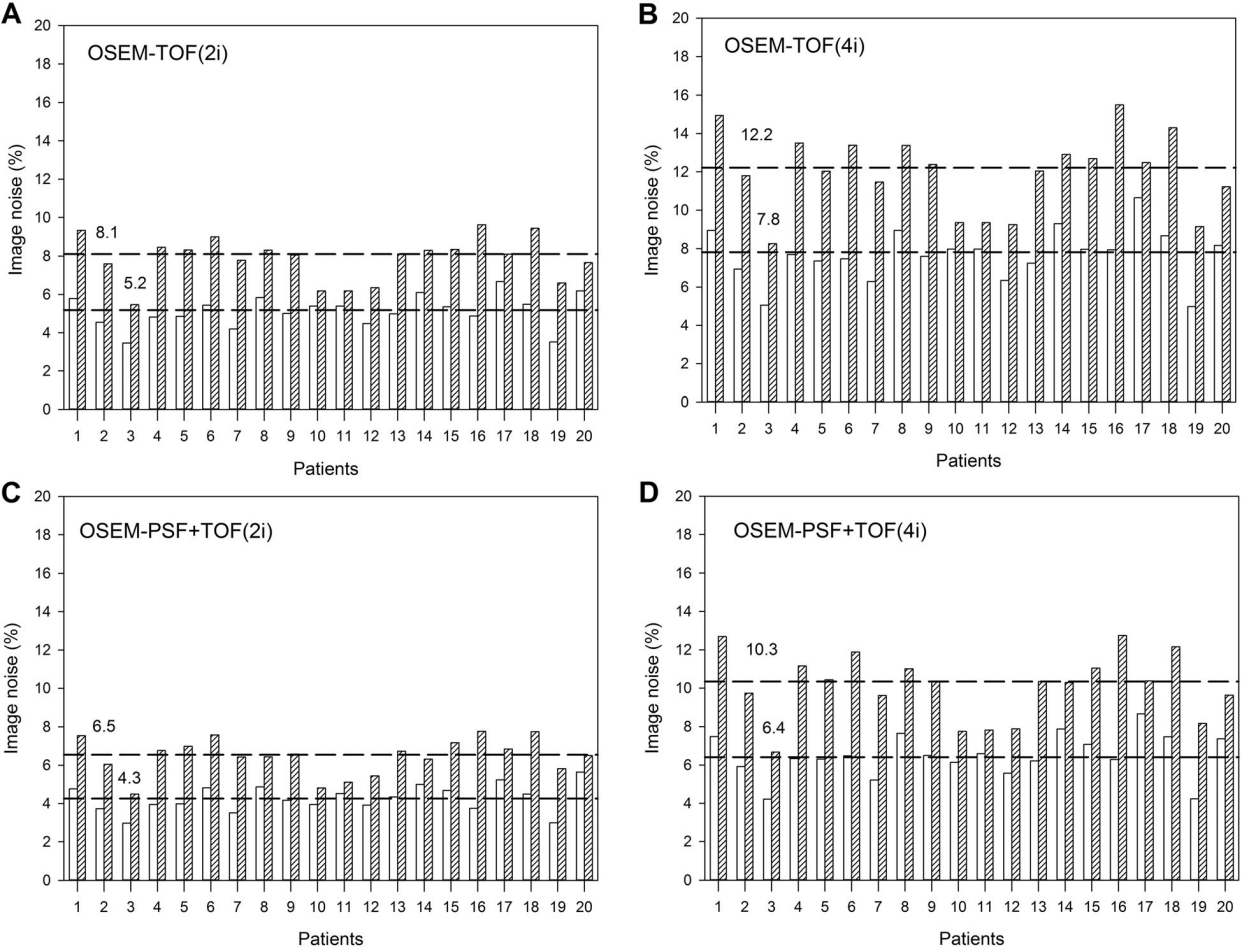
Bar graph showing the image noise derived from the liver’s activity distribution for images acquired with reference (white bars) vs. reduced acquisition time (hatched bars) across all reconstruction algorithms. Median values for each reconstruction algorithm are given and they are indicated with dashed lines

Scan time reduction led to an increase in image noise and these differences were most pronounced in the images reconstructed with 4 iterations (Fig. 3). The mean image noise increased from 7.8 to 12.2% for OSEM-TOF (4i) and 5.2 to 8.1% for OSEM-TOF (2i). The same phenomenon was observed for OSEM-PSF+TOF reconstructed images, that is, image noise increased from 6.4 to 10.3% OSEM-TOF+PSF (4i), 4.3 to 6.5% for OSEM-TOF+PSF (2i)

## Discussion

This study confirms that a digital PET/CT system enables a reduction of scan time duration or administered ^18^F-FDG-PET/CT activity. In our cohort of 20 lymphoma patients all of our defined body regions (supradiaphragmal nodal, infradiaphragmal nodal, extranodal) were correctly classified and no down-staging was observed using images acquired with the reduced acquisition protocol. Hence, based on the images acquired with almost a threefold reduction in scan time duration, lesion detectability, image quantification, and image noise were highly reproducible across all reconstruction algorithms.

Similarly, van Sluis et al. [10] were able to show that a threefold reduction in scan time duration led to down-staging in a minor fraction of patients (1/30) of their patient cohort that encompassed different oncological entities. A prior study by our group showed comparable results for ^68^Ga-PSMA PET in prostate cancer [18]. We observed down-staging in 1/20 patients due to missing small nodal lesions. The differences in findings to the previous study can be explained by the bigger size of nodal lesions in this study (mean short-axis diameter: 13.8 mm) as well as the different imaging properties of ^18^F-FDG-based tracers [19]. However, at presentation, size of nodal lesions in lymphoma is often larger than 1 cm, so the lesion size in our patient cohort is likely to be representative [20]. Figure 1 shows an exemplary patient, who underwent ^18^F-FDG-PET/CT using the reference acquisition and reduced acquisition protocol.

Image quantification acquired with the reduced scan time duration proved to be reliable across all reconstruction algorithms and the single absolute percentage deviation was considerably lower than < 20% (Fig. 2), which we previously defined as the acceptable margin of error. Of note, a recent study by Kurland et al. [17] demonstrated that lesion uptake (SUVmax) showed an average difference of 8% for the same scanner model within the same institution and 18% for different scanners from different institutions. No notable differences were observed between SUVmax and SUVpeak measurements and between low, moderate and high tracer uptake. In this study images acquired with the reduced acquisition protocol were acquired after the reference protocol. The occurrence of at least slight metabolic changes between the acquisitions of both scans is likely as prior studies employing dual time point ^18^F-FDG-PET/CT have shown an increase of tumor specific ^18^F-FDG-uptake on images acquired as late as 2 or 3 h after injection [21, 22]. This might partially explain the higher tumoral uptake on images acquired with reduced vs. reference emission time. A detailed depiction of quantitative assessment in all patients is provided in Fig. 2.

As expected, a reduction of emission time led to an increase in image noise, which is in line with prior studies by van Sluis et al. [10] and Sonni et al. [23]. An in-depth overview of image noise across all employed reconstruction algorithms is provided in Fig. 3.

Interestingly, a higher image noise was observed for images reconstructed with 2 iterations vs. 4 iterations, which is comparable with a previous study of our group on emission time reduction in ^68^Ga-PSMA PET/CT [18].

Limitations of our study are the relatively small patient and lesion size and its retrospective nature. Additionally, in more than half of patients ^18^F-FDG-PET/CT did not reveal any ^18^F-FDG-avid lymphoma manifestations, which further restricts the reliability of our study results. For ethical reasons histopathological lesion validation was not performed. However, this was beyond the scope of this study as ^18^F-FDG-PET/CT constitutes the current imaging gold standard [24].

## Conclusion

This study shows that the advent of the new generation of digital PET/CT systems might enable a reduction of scan time duration (or administered activity) without sacrificing diagnostic performance. Especially a reduction in tracer activity might allow for higher patient throughput, better cost-efficiency, and a reduction in radiation exposure in the frequently young lymphoma patients. However, the results have to be validated in larger trials.

## Supplementary Information

**Additional file 1: Table S1.** Patient and Imaging Characteristics (*n*=20).

**Additional file 2: Table S2.** Overview of the Lesion detectability using images reconstructed with OSEM-TOF+PSF 4i (served as reference) in comparison with the detectability using images reconstructed with OSEM-TOF 4i (short) and OSEM-TOF 2i (reduced).

**Additional file 3: Table S3.** Overview of the lesion detectability using images reconstructed with OSEM-TOF 4i (served as reference) in comparison with the detectability using images reconstructed with OSEM-TOF 4i (short) and OSEM-TOF 2i (reduced).

## Data Availability

The datasets generated and/or analysed during the current study are not publicly available due to privacy legislation but are available from the corresponding author on reasonable request.

## Abbreviations

^18^F-FDG PET/CT: Fluorodeoxyglucose
PET: positron-emission tomography
CT: computed tomography
TOF: time of flight
PSF: point spread function
EANM: European Association of Nuclear Medicine
MBq: Megabecquerel
SD: standard deviation
kV: kilovolts
FOV: field of view
OSEM: Poisson ordered-subset expectation maximization
4i: 4 iterations
2i: 2 iterations
SUVmax: maximum standardized uptake value
SUVpeak: peak standardized uptake value
