# Supplementary material for: Evaluation of 18F-FDG PET/CT images acquired with a reduced scan time duration in lymphoma patients using the digital biograph vision
Source: BMC Cancer. 2021 Jan 14;21:62. doi: 10.1186/s12885-020-07723-2 (PMC7807699; doi:10.1186/s12885-020-07723-2)
Supplement: Supplementary file 1 — Additional file 1: Table S1. Patient and Imaging Characteristics (n=20). [file 12885_2020_7723_MOESM1_ESM.docx]

| Table S1. Patient and Imaging Characteristics (n=20) | |
| --- | --- |
| Sex and age | Values for age |
| Mean (range) age (in years) | 50.0 (23–84) |
| Mean (range) weight (in kg) | 81 (47–130) |
| Men | 8 |
| Women | 12 |
| Lymphoma subtype | Number of patients |
| Hodgkin lymphoma  High clinical suspicion, NOS  NHL  - DLBCL  - Follicular lymphoma  - ALCL  - BCL NOS  - NHL NOS | 5  1  14  - 6  - 2  - 1  - 3  - 2 |
| Indication | Number of patients |
| Initial staging  Assessment of treatment response  Suspicion for recurrence | 6  12  2 |
| Imaging characteristics |  |
| Mean administered activity ±SD  Mean uptake time ± standard deviation | 340±72  73±11 |

NOS: Non otherwise specified; NHL: Non-Hodgkin lymphoma; DLBCL: Diffuse B-Cell

Lymphoma; ALCL: anaplastic large-cell lymphoma; BCL: B-cell lymphoma, NHL: Non-

Hodgkin lymphoma
