# Supplementary material for: Evaluation of 18F-FDG PET/CT images acquired with a reduced scan time duration in lymphoma patients using the digital biograph vision
Source: BMC Cancer. 2021 Jan 14;21:62. doi: 10.1186/s12885-020-07723-2 (PMC7807699; doi:10.1186/s12885-020-07723-2)
Supplement: Supplementary file 2 — Additional file 2: Table S2. Overview of the Lesion detectability using images reconstructed with OSEM-TOF+PSF 4i (served as reference) in comparison with the detectability using images reconstructed with OSEM-TOF 4i (short) and OSEM-TOF 2i (reduced). [file 12885_2020_7723_MOESM2_ESM.docx]

Table S2 Overview of the Lesion detectability using images reconstructed with OSEM-TOF+PSF 4i (served as reference) in comparison with the detectability using images reconstructed with OSEM-TOF 4i (short) and OSEM-TOF 2i (reduced).

| Patient No. | OSEM-PSF+TOF 4i (reference) | OSEM-PSF+TOF 4i (reduced) | OSEM-PSF+TOF 2i (reduced) |
| --- | --- | --- | --- |
| 5 | 1/0/0 (II) | 1/0/0 (II) | 1/0/0 (II) |
| 9 | 0/0/0 (0) | 0/0/0 (0) | 0/0/0 (0) |
| 10 | 0/0/1 (IV) | 0/0/1 (IV) | 0/0/1 (IV) |
| 11 | 0/0/0 (0) | 0/0/0 (0) | 0/0/0 (0) |
| 12 | 0/0/1 (I) | 0/0/1 (I) | 0/0/1 (I) |
| 13 | 0/0/0 (0) | 0/0/0 (0) | 0/0/0 (0) |
| 14 | 1/1/0 (III) | 1/1/0 (III) | 1/1/0 (III) |
| 15 | 0/0/0 (0) | 0/0/0 (0) | 0/0/0 (0) |
| 16 | 0/0/0 (0) | 0/0/0 (0) | 0/0/0 (0) |
| 17 | 0/0/0 (0) | 0/0/0 (0) | 0/0/0 (0) |
| 18 | 1/0/1 (IV) | 1/0/1 (IV) | 1/0/1 (IV) |
| 19 | 0/0/0 (0) | 0/0/0 (0) | 0/0/0 (0) |
| 20 | 1/1/0 (III) | 1/1/0 (III) | 1/1/0 (III) |
| 21 | 1/0/0 (I) | 1/0/0 (I) | 1/0/0 (I) |
| 22 | 0/0/0 (0) | 0/0/0 (0) | 0/0/0 (0) |
| 23 | 0/0/0 (0) | 0/0/0 (0) | 0/0/0 (0) |
| 24 | 0/1/0 (II) | 0/1/0 (II) | 0/1/0 (II) |
| 25 | 1/0/0 (II) | 1/0/0 (II) | 1/0/0 (II) |
| 26 | 0/0/0 (0) | 0/0/0 (0) | 0/0/0 (0) |
| 27 | 0/0/0 (0) | 0/0/0 (0) | 0/0/0 (0) |

Supradiaphragmal positive? / infradiaphragmal positive? / Extranodal positive? (Ann-Arbor-Stage); „1“: yes, „0“: no
